# A Bayesian Hierarchical Approach for Relating PM_2.5_ Exposure to Cardiovascular Mortality in North Carolina

**DOI:** 10.1289/ehp.6980

**Published:** 2004-06-03

**Authors:** Christopher H. Holloman, Steven M. Bortnick, Michele Morara, Warren J. Strauss, Catherine A. Calder

**Affiliations:** ^1^Statistics and Data Analysis Systems, Battelle Memorial Institute, Columbus, Ohio, USA; ^2^Department of Statistics, The Ohio State University, Columbus, Ohio, USA

**Keywords:** exposure simulator, fine particulate matter, SHEDS-PM, spatial modeling, Stochastic Human Exposure and Dose Simulation

## Abstract

Considerable attention has been given to the relationship between levels of fine particulate matter (particulate matter ≤ 2.5 μm in aerodynamic diameter; PM_2.5_) in the atmosphere and health effects in human populations. Since the U.S. Environmental Protection Agency began widespread monitoring of PM_2.5_ levels in 1999, the epidemiologic community has performed numerous observational studies modeling mortality and morbidity responses to PM_2.5_ levels using Poisson generalized additive models (GAMs). Although these models are useful for relating ambient PM_2.5_ levels to mortality, they cannot directly measure the strength of the effect of exposure to PM_2.5_ on mortality. In order to assess this effect, we propose a three-stage Bayesian hierarchical model as an alternative to the classical Poisson GAM. Fitting our model to data collected in seven North Carolina counties from 1999 through 2001, we found that an increase in PM_2.5_ exposure is linked to increased risk of cardiovascular mortality in the same day and next 2 days. Specifically, a 10-μg/m^3^ increase in average PM_2.5_ exposure is associated with a 2.5% increase in the relative risk of current-day cardiovascular mortality, a 4.0% increase in the relative risk of cardiovascular mortality the next day, and an 11.4% increase in the relative risk of cardiovascular mortality 2 days later. Because of the small sample size of our study, only the third effect was found to have > 95% posterior probability of being > 0. In addition, we compared the results obtained from our model to those obtained by applying frequentist (or classical, repeated sampling-based) and Bayesian versions of the classical Poisson GAM to our study population.

Researchers have found that acute episodes of increased particulate matter (PM) are associated with nonaccidental mortality (Goldberg et al. 2001), total mortality (Katsouyanni et al. 2001; Laden et al. 2000; Mar et al. 2000; Wichmann et al. 2000), cardiovascular deaths (Hoek et al. 2001; Ostro et al. 2000), respiratory deaths (Braga et al. 2001; Hoek et al. 2001), elderly deaths (Katsouyanni et al. 2001), asthma in children and the nonelderly (Lin et al. 2002; Norris et al. 1999; Sheppard et al. 1999), and morbidity (Schwartz 1999; Zanobetti et al. 2000). In all of these studies, the approach taken by the researchers to establish a connection between ambient PM levels and health end points consists of relating measured PM levels on a given day to mortality or morbidity rates on the same or closely following days while adjusting for possible confounding factors such as weather, day of the week, and long-term trends in mortality rates. By far, the most common model used to establish this relationship is the Poisson generalized additive model (GAM). Poisson GAMs are well suited for addressing the question of whether levels of ambient PM in the outdoor environment are associated with health end points, but they may not be the best approach for quantifying the relationship between PM exposure and health end points because direct exposure data cannot be collected for large populations over long periods of time. As a result, Poisson GAMs cannot give direct estimates of increases in the relative risk of morbidity and mortality as a result of exposure to PM.

In attempting to explore the relationship between PM exposure and morbidity or mortality, care should be taken not to assume that the relationship between ambient levels and mortality implies a similar connection between exposure and mortality. It is well documented that ambient levels poorly approximate true exposure (Dockery and Spengler 1981; Lioy et al. 1990; Spengler et al. 1985; Tamura and Ando, unpublished data), and ignoring the discrepancy between exposure and ambient levels in investigations of health effects can lead to biases and underestimation or overestimation of the uncertainty about effects even in simple models (Armstrong et al. 1992). One recent study from the Health Effects Institute (HEI; Cambridge, MA) shows that PM studies are no different: ignoring exposure information can result in biases and misrepresentation of uncertainty when linking PM to health effects (Samet et al. 2000).

In an effort to include exposure information in a model linking levels of PM ≤ 10 μm in aerodynamic diameter (PM_10_) and mortality, an HEI study (Samet et al. 2000) proposed a multistage Bayesian Poisson regression model, a generalization of the GAM, that includes exposure information. The focus of the HEI study was on Baltimore, Maryland, where daily mortality, PM_10_, and weather variables were collected from 1987 through 1994. Within Baltimore, Samet et al. used the Poisson GAM form to relate PM_10_ exposure (instead of ambient levels) to mortality. At the next stage of the hierarchy, the latent exposure is related to ambient PM levels using a linear regression form. To provide information about the coefficients of the regression relating the latent exposure to ambient levels, Samet et al. hypothesized that the same linear form is appropriate for each of five exposure studies and linked the coefficients in each study and the Baltimore population together through another level in the hierarchy.

Although the approach of Samet et al. (2000) takes an important step forward by including exposure information in an epidemiologic model, the method of relating ambient levels to exposure levels could be improved. The assumption that the linear relationship between PM_10_ levels and true exposure is similar between the Baltimore population and the populations in the five exposure studies may be unwarranted. In contrast to this HEI approach, an alternative approach for relating ambient pollutant levels to true personal exposure that has gained acceptance more recently is the use of computer exposure simulators. Zidek et al. (2003) presented a general statistical framework for the construction of these simulators. Exposure simulators use activity data and microenvironment pollutant-level data to estimate pollutant exposure levels for individuals. One of the most sophisticated exposure simulators to date for PM is the Stochastic Human Exposure and Dose Simulation (SHEDS-PM) (Burke et al. 2001). For a single individual, SHEDS-PM stochastically simulates a PM level for each of the environments in which the individual spends time. Once SHEDS-PM has defined the microenvironmental levels, the total PM exposure for the individual is estimated by weighting the PM levels in the various environments by the amount of time the individual spends in each of those environments. By examining the estimated PM exposure levels of several individuals created in this manner, the distribution of exposure levels for a population can be characterized.

Building upon the Bayesian model used in the HEI study (Samet et al. 2000), we propose a Bayesian hierarchical model for modeling the relationships among levels of ambient fine PM (particulate matter ≤ 2.5 μm in aerodynamic diameter; PM_2.5_), average exposure to PM_2.5_, and cardiovascular mortality that incorporates an exposure simulator similar to SHEDS-PM. Unlike most studies, our model allows us to directly quantify the effect of exposure to PM_2.5_ on cardiovascular mortality. Bayesian hierarchical modeling is a framework that allows multiple data sources and statistical modeling techniques to be incorporated into a single coherent statistical model (Gelman et al. 1995). In contrast to the Poisson GAM, our model describes the hierarchical nature of the process that connects monitor readings of PM_2.5_ to cardiovascular mortality by using a three-level hierarchy. The hierarchy is summarized in Table 1. At the first level, we describe the relationship between PM_2.5_ monitors and a continuous surface of ambient PM_2.5_ concentrations by allowing for monitor error and considering the spatial properties of PM_2.5_. At the next level, we link average ambient PM_2.5_ concentrations at the county level to average population exposure at the county level using an exposure simulator similar to SHEDS-PM. Finally, the third level links average exposure levels to daily cardiovascular mortality counts using the Poisson GAM form. By incorporating all of these levels into a single Bayesian hierarchical model, we are able to estimate the effect of PM_2.5_ exposure on cardiovascular mortality and to combine several disparate sources of data in a meaningful way. Although not clearly marked in Table 1, note that the modeled process from level 1 feeds into the modeling technique for level 2, and the modeled process from level 2 feeds into the modeling technique for level 3. By fitting our model using 3 years of data in seven counties in North Carolina (Alamance, Chatham, Durham, Guilford, Johnston, Randolph, and Wake), we found that increased PM_2.5_ exposure is related to increased risk of cardiovascular mortality on the same day and the next 2 days. The size of the observed effect is greater than that observed between ambient PM_2.5_ levels and cardiovascular mortality, although similar patterns in the effects appear.

## Materials and Methods

Mortality data for North Carolina for the years 1999–2001 were obtained from the website of the Odum Institute at the University of North Carolina (Odum Institute 2003). These data were subdivided to include only deaths from cardiovascular causes [*International Classification of Diseases, 10th Revision* (ICD-10) codes I00 to I99; World Health Organization (WHO) 1992]. PM_2.5_ data for all available monitors in North Carolina during 1999–2001 were obtained from the U.S. Environmental Protection Agency (EPA) Aerometric Information Retrieval System/Air Quality Subsystem (AIRS/AQS) database (U.S. EPA 2003b). Each monitor in North Carolina takes readings on a daily, 1-in-3-day, or 1-in-6-day schedule. Daily meteorologic data across North Carolina were obtained from the National Oceanographic and Atmospheric Association’s (NOAA) National Climatic Data Center (Asheville NC) via online subscription (NOAA 2003). For each county, the values of the three variables of interest (daily maximum temperature, average wind speed, and relative humidity) were assumed to be equal to the values of those variables reported by the weather station closest to the centroid of the county. We imputed missing meteorologic data (~ 2% missing overall) by calculating the average value for all other counties with complete data on the same day and substituting that average value for the missing value. Data on human activity patterns were obtained from the Consolidated Human Activities Database (CHAD; U.S. EPA 2003a). This database contains the results of 12 studies in which individual 24-hr details of activities and the environments in which those activities took place were recorded. We restricted our use of the database to records contained in the National Human Activity Pattern Survey (NHAPS) portion of the CHAD and to records of individuals > 20 years of age. Demographic data on the county level were obtained from the U.S. Census Bureau (2003). The population counts for the 2000 census were assumed to be representative of the population counts across the time period studied (1999–2001). We used two level-3 summary files in our analysis, P1 and PCT35, which include total population counts by county and the number of individuals > 16 years of age in each county by sex, age, and employment status, respectively.

The model that we propose for relating PM_2.5_ readings at monitors to daily cardiovascular mortality counts is a three-level hierarchical Bayesian model. The three levels in our model are as follows: *a*) linking monitor readings to ambient levels over the study region, *b*) linking ambient levels to exposure levels, and *c*) linking exposure levels to mortality (Table 1).

### Level 1.

Central to our model relating PM levels to mortality is that, for any given day, a continuous surface of ambient PM_2.5_ levels exists over the study region; this is what would be measured if we obtained an infinite number of monitor readings (spatially dense) without error each day. The first level of our model specifies the spatial distribution of PM_2.5_ and relates that distribution to readings taken at monitors on a single day.

We conducted a spatial analysis of PM_2.5_ and determined that PM_2.5_ exhibits strong spatial correlation over the region of interest [details reported by Calder et al. (2003)]. In order to incorporate this information into a statistical model, we assigned a joint multivariate normal distribution to any set of observations of the PM_2.5_ surface. Although we acknowledge that PM_2.5_ readings tend to be right-skewed rather than normally distributed, this simplification is not expected to have a strong impact on the overall model fit and simplifies model fitting considerably. On any day *t* and for any set of sites *s*(1), … , *s*(*n*_ψ_), the distribution of the PM_2.5_ surface ψ*_t_* at those points is ψ*_t_* | θ ~ MN*_n_*_ψ_ (*M_t_*θ, ∑), where ψ*_t_* = [ψ*_t_*(*s*_1_) … , ψ*_t_*[*s*(*_n_*_ψ_)]*^T^*, MN is the multivariate normal distribution, *M_t_* is a design matrix of covariates, θ is a parameter vector, and ∑ is an *n*_ψ_ × *n*_ψ_ spatial covariance matrix constructed using information from our exploratory spatial analysis of outdoor PM_2.5_ levels. For each site, *s*(1), … , *s*(*n*_ψ_), *M_t_* includes a row with elements representing an overall mean, maximum temperature, average wind speed, and two sinusoidal terms that capture seasonal cycles. We considered the corresponding five regression coefficients, θ = (θ_0_, … , θ_4_), to be unknown, and we minimized prior influence by placing vague *N*(0, 100) priors on these parameters.

The sites *s*(1), … , *s*(*n*_ψ_) for which the spatial distribution of PM_2.5_ is estimated need not be locations with monitors. The matrices *M_t_* and ∑ are defined for any location in our modeled domain. In fact, in our implementation we modeled the spatial process at several locations that do not have monitors to better characterize the average ambient level over the entire spatial area of each county.

In relating monitor readings to the ambient surface we have defined, we assumed that the PM_2.5_ monitors measure the ambient PM_2.5_ surface with some error (measurement error and other random sources of error) at their locations: *X_t_*(*s*) | ψ*_t_*(*s*), σ*_x_*^2^ ~ *N*[ψ*_t_*(*s*), σ*_x_*^2^], where *X_t_*(*s*) is the monitor reading at monitoring site *s* at time *t*, ψ*_t_*(*s*) is the value of the ambient surface at the location of monitoring site *s* at time *t*, and σ*_x_*^2^ is the variance of the measurement error. This construction automatically incorporates the additional uncertainty about the ambient PM_2.5_ surface on days when fewer monitors take readings. Days when more monitors take readings (every third or sixth day) will carry more information about the ambient surface than will days when only a subset of daily monitors takes readings, so our uncertainty about the ambient surface will be smaller on these days.

In order to construct a prior distribution for σ*_x_*^2^, the variance of the measurement error at the PM_2.5_ monitors, precision and accuracy data were downloaded from the AIRS/AQS database (U.S. EPA 2003b). Using these data, we developed an inverse-gamma (649, 1433.405) prior distribution (mean = 2.2, variance = 7.5 × 10^−3^) for σ*_x_*^2^. This prior was developed using a simple conjugate inverse-gamma/normal model [e.g., Gelman et al. (1995)] with an inverse-gamma (1, 1) prior on σ*_x_*^2^ before observing data.

By creating a continuous surface of ambient PM_2.5_ levels, we gained several advantages over the more common “monitor averaging” approach. First, information on the ambient PM_2.5_ level on any given day is shared across counties, allowing more accurate characterization of ambient levels in all locations. Second, the interpolation of a continuous ambient surface allows inference about the ambient level in counties that do not contain any PM_2.5_ monitors, thereby giving better representation to rural counties. Third, the Bayesian specification of the prior distribution on the ambient level allows natural incorporation of seasonal cycles and meteorologic effects on PM_2.5_ levels. Finally, we can characterize the average ambient level in any county on any day by averaging the spatial surface over the county.

### Level 2.

Level 2 of our model links average ambient PM_2.5_ levels in a county to the average exposure level within that county. In this level of the model, we used a deterministic population-level exposure simulator to assist in relating ambient levels to true exposure. Our simulator uses human activity data, information about PM_2.5_ levels in indoor environments, and the average ambient concentration on a given day to approximate the exposure level of several individuals in a county on that day. Then, the exposure levels for these individuals are averaged to estimate an average exposure level for all individuals in the county on that day. The population-level exposure simulator used in our model is an adaptation of the SHEDS-PM simulator proposed by Burke et al. (2001). Like SHEDS-PM, our simulator calculates exposure for an individual person using an activity diary and ambient PM_2.5_ levels as inputs. This process is repeated for several individuals, and the resulting average exposure is estimated as the mean of the individual exposure levels.

Assuming that the outdoor PM_2.5_ level is known and the activity pattern of an individual is known, our simulator calculates individual exposure as follows:


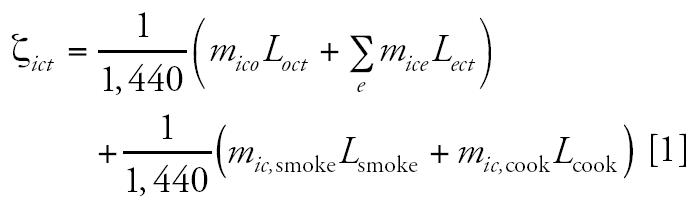


where ζ*_ict_* is the exposure level for individual *i* in county *c* on day *t*, *m_ico_* is the number of minutes the individual spends outdoors, *m_ice_* is the number of minutes the individual spends in indoor microenvironment *e* (residential, office, school, store, vehicle, restaurant, and bar), *m_ic_*_,smoke_ is the number of minutes the individual spends with smokers present, *m_ic_*_,cook_ is the number of minutes the individual spends cooking, *L_oct_* is the ambient PM_2.5_ level in county *c* on day *t*, *L_ect_* is the PM_2.5_ level in indoor microenvironment *e* in county *c* on day *t*, *L*_smoke_ is the addition to the PM_2.5_ level in the current microenvironment when smokers are present, *L*_cook_ is the addition to the PM_2.5_ level in the current microenvironment when the individual is cooking, and 1,440 is the number of minutes in a day. When the simulator is implemented in our statistical model, *L_oct_* is set equal to the average ambient level in the county at time *t*, ψ̄*_ct_*. Additional PM_2.5_ measures from smoking and cooking are fixed at 10 μg/m^3^ [based on values reported by Burke et al. (2001)] and 5 μg/m^3^ [based on findings of Wallace et al. (2003)]. We kept these values constant to simplify computation; a more accurate approach would be to account for the brief shock these activities give to indoor PM_2.5_ levels stochastically. Note that this equation makes no distinction between the toxicity of indoor and outdoor particles in our model. The values of *L_ect_* for indoor microenvironments are calculated as linear functions of the outdoor level: *L_ect_* = *a_e_* + *b_e_L_oct_* for *e* in the set {residential, office, school, store, vehicle, restaurant, bar}. Values of *a_e_* and *b_e_* are shown in Table 2. These values were calculated using simplifications of values reported by Burke et al. (2001) for SHEDS-PM.

In each of the counties in which we hope to model the relationship between exposure and cardiovascular mortality, we applied the exposure simulator to several individuals to estimate an average exposure value. In order to apply the simulator, we used activity data that are representative of the true activity patterns in each county in which we modeled the mortality/exposure link. We simulated the activity data by randomly sampling 100 individuals from the county of interest using census demographic information (U.S. Census Bureau 2003) and matching each individual with an activity record from the CHAD (U.S. EPA 2003a). These activity records are drawn from diaries kept across the entire country. Despite possible geographic mismatches, this method of obtaining activity information is usually sufficient for obtaining representative activity information (Özkaynak H, personal communication). To simplify model implementation, a single activity pattern was associated with each individual, and no adjustments were made for different times of the year (i.e., winter vs. summer activity patterns).

To account for possible discrepancy between the simulator predicted value of exposure and true exposure levels, we specified that the average exposure level in a given county is normally distributed around the –value predicted by the simulator: *Z_ct_* | ψ̄*_ct_*, σ*_z_*^2^ ~ *N*[ξ(ψ̄*_ct_*), σ*_z_*^2^], where *Z_ct_* is the average exposure level in county *c* at time *t*, ψ̄*_ct_* is the average ambient level in county *c* at time *t*, ξ(ψ̄*_ct_*) is the average exposure level predicted by the simulator in county *c* at time *t* as a function of the average ambient level, and σ*_z_*^2^ is the variance of the error in the simulator. We place a uniform (0, 25) prior on σ*_z_*^2^. Although there is not enough information in the data to estimate σ*_z_*^2^ accurately, allowing it to be random incorporates our uncertainty in the simulator into the model resulting in more accurate uncertainty estimates at the third level.

### Level 3.

In the third level of the model, we linked exposure directly to mortality using the Poisson GAM form commonly used in studies of the link between PM_2.5_ and mortality. Mortality was assumed to be Poisson distributed with a mean that depends on average PM_2.5_ exposure in the current and 3 previous days as well as the values of several confounders:


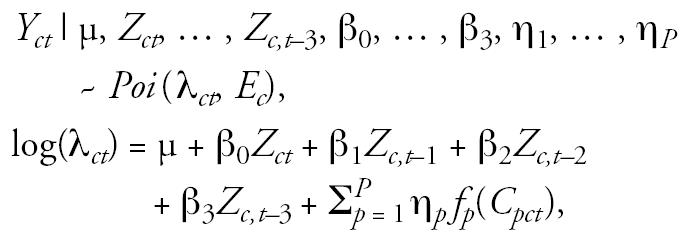


where *Y_ct_* is the mortality in county *c* on day *t*, *E_c_* is the expected daily mortality rate in county *c* (necessary for adjusting the mean level so that the β and η parameters have the same interpretation in all counties), λ*_ct_* may be interpreted as a relative risk of death in county *c* on day *t*, μ is an overall baseline relative risk of death in the study region over the time period studied, β_0_, … , β_3_ are parameters describing the influence of county-level average exposure on mortality rate, *f_p_*(*C_pct_*) are transformations of confounding variables, and η_1_, … , η*_P_* are parameters describing the influence of confounding variables on mortality. For our data set, confounding variables included a factor variable for the day of the week, a cubic spline transformation of time to account for long-term trends in cardiovascular mortality, a cubic spline transformation of maximum temperature, a cubic spline transformation of relative humidity, and cubic spline transformations of 1- to 3-day lagged values of maximum temperature and relative humidity. The cubic spline transformation of time included 21 evenly spaced knots, and the cubic spline transformations of maximum temperature and relative humidity each included five evenly spaced knots. The model was not assessed for sensitivity to the placement of these knot locations. We reparameterized the confounding variable term into a design matrix (&*C*tilde;) and coefficient vector (γ), and we placed vague *N*(0, 100) priors on the coefficients. We also placed vague *N*(0, 100) priors on all of the β-parameters describing the strength of the relationship between PM_2.5_ exposure and cardiovascular mortality at different lags as well as on the overall mean relative risk parameter, μ.

### Summary.

Although we have introduced a three-level model, we emphasize that the three levels of the model are all fitted simultaneously as a single coherent statistical model. There are three main advantages to creating a hierarchical Bayesian model for solving such a complex problem. The most important advantage is that uncertainty in parameters is propagated throughout the model. For example, our uncertainty about the true ambient surface (due to errors in the monitors and the necessity of spatial interpolation) carries through to result in a corresponding level of uncertainty about the effect of exposure on cardiovascular mortality. The second important advantage of hierarchical Bayesian modeling is that it is simple to specify large, complex models using simpler statements about conditionally independent parameters. It would be impossible to specify the joint distribution of the thousands of parameters involved in our model if we tried to model the spatial properties of PM_2.5_, the relationship between exposure and ambient levels, and the relationship between exposure and cardiovascular mortality simultaneously. In contrast, the hierarchical approach allows us to specify each level of the model conditionally independent of other levels and to combine the information at the end to obtain a joint distribution of all parameters. The third advantage is that elements of the hierarchy can be substituted without changing the overall form of the model. For instance, we could substitute a different exposure simulator in the second level of the model.

## Results

Model fitting was performed using a Markov chain Monte Carlo algorithm (Gelfand and Smith 1990; Geman and Geman 1984; Hastings 1970). The algorithm was implemented with custom C++ software developed using Microsoft Visual Studio (Microsoft Corporation, Redmond, WA). Random number generation was performed using functions from the Numerical Algorithms Group library (NAG, Ltd, Oxford, UK). The algorithm was run for 200,000 iterations, 50,000 of which were discarded as “burn-in” iterations. To reduce the storage space for the samples, the remaining 150,000 samples were thinned by a factor of 50, resulting in a total of 3,000 draws from the joint posterior distribution.

The marginal posterior distributions of several important parameters are summarized in Table 3. For each of the parameters, we include an estimate of the posterior mean (calculated by averaging samples from the posterior distribution) and posterior median (calculated as the median of the sample), a Monte Carlo error for the mean, and a posterior 95% credible interval. The Monte Carlo error for the mean describes how far off our estimate of the true posterior mean is as a result of using a Monte Carlo method for exploring the posterior; it does not describe the uncertainty in the actual parameter. The 95% credible interval does describe the uncertainty in the parameter; it is an equaltail interval such that the posterior probability that the parameter falls within the interval is 95%. Credible intervals are the Bayesian analogue of the confidence interval but are much easier to interpret because they give direct information about the probability of a parameter falling within certain bounds.

The posterior analysis indicates a positive effect of PM_2.5_ exposure on the relative risk of cardiovascular mortality. The posterior marginal expectations of the parameters indicate that a 10-μg/m^3^ increase in average PM_2.5_ exposure is associated with a 2.5% increase (95% credible interval, –3.9 to 9.6) in the relative risk of current day cardiovascular mortality, a 4.0% increase (–3.3 to 12.2) in the relative risk of cardiovascular mortality the next day, an 11.4% increase (2.8 to 19.8) in the relative risk of cardiovascular mortality 2 days later, and a 1.1% decrease (–7.5 to 5.2) in the relative risk of cardiovascular mortality 3 days later. These rates were calculated by multiplying the β-value corresponding to the effect by 10 and exponentiating. Only the effect on the second day after exposure has a > 95% posterior probability of exceeding zero. Note that the estimates presented are marginal expectations and therefore cannot be added together (e.g., to get an overall risk of cardiovascular mortality from exposure to PM_2.5_) in a meaningful way. The negative estimate on the third day might be considered an unexpected effect, but it does lend some support to the theory of harvesting (Schwartz 2000). This theory hypothesizes that individuals close to dying of cardiovascular-related causes may die soon after a spike in PM_2.5_ exposure, leaving only healthier individuals and consequently decreasing the overall risk of cardiovascular mortality in the total population.

We are unaware of any other study that has attempted to directly estimate the effect of PM_2.5_ exposure on mortality, but some related estimates for PM_10_ are available from the HEI study (Samet et al. 2000). In that study, a 10-μg/m^3^ increase in PM_10_ exposure is associated with a 1.4% increase in same-day relative risk of mortality. Although the uncertainty about the HEI estimate is much smaller (probably as the result of a longer time period of study), the point estimate is similar to the one obtained in our analysis.

**Figure 1:**
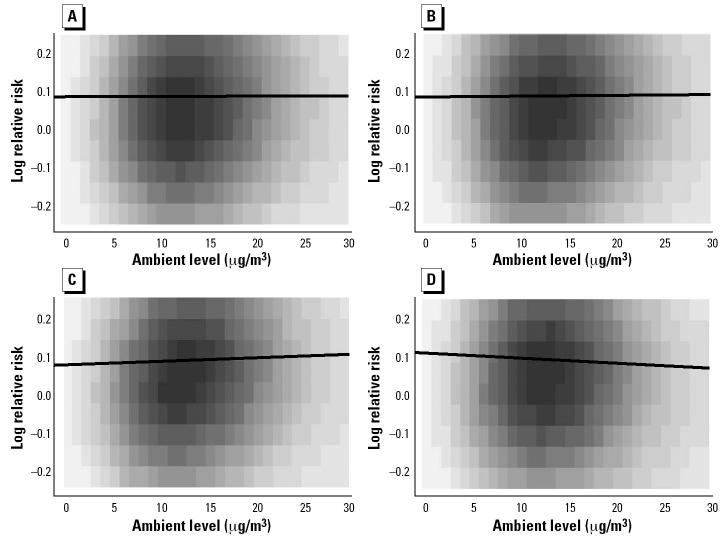
Joint distribution of ambient PM_2.5_ level and log relative risk on the same day (*A*), the next day (*B*), 2 days later (*C*), and 3 days later (*D*), with lines summarizing the direction of association (described in ”Results”). Darker areas represent regions of higher probability. The exponential of the slope of the line in each panel represents the proportion change in relative risk per unit change in ambient level.

Although our main goal in this analysis was to demonstrate the effect of PM_2.5_ exposure on cardiovascular mortality, we can also address the effect of changes in the ambient level on the relative risk of cardiovascular mortality. To determine the relationship between ambient levels and relative risk induced by our model, we examined the joint posterior distribution of average ambient levels, ψ̄*_ct_*, and log relative risk, λ*_ct_*, on the same and closely following days. Figure 1 shows smoothed images of the joint distributions combining information across counties. Lines have been added to the figures to illustrate the overall direction of the effect; the line is chosen to minimize the sum of squared distances between samples from the distribution (not shown) and the line. The slope of the line is a summary of the effect of an increase in average ambient level on the log relative risk of cardiovascular mortality, although it is not a parameter in the model. By exponentiating the slope of the line, we obtain an estimate of the proportional increase in relative risk associated with a unit change in ambient level. The lines imply that a 10-μg/m^3^ increase in ambient level is associated with a 0.09% increase in the relative risk of cardiovascular mortality on the same day, a 0.2% increase the next day, a 1.0% increase 2 days later, and a 1.4% decrease 3 days later. As with the estimates of effect of exposure on cardiovascular mortality, these estimates are marginal effects and should be interpreted individually; they should not be combined to find an overall effect. These estimates tend to be lower than some comparable estimates reported in the epidemiologic literature. The effect of 2-day mean ambient levels on total mortality has been estimated at 3.3% for chronic obstructive pulmonary disease, 2.1% for ischemic heart disease [both estimates from Schwartz et al. (1996)], and 1.5% for total mortality from natural causes (Klemm et al. 2000), all higher than our largest estimate. This result is not surprising because the inclusion of an exposure link in our model should weaken the direct relationship between ambient levels and mortality. The trend of a weaker association between ambient levels and mortality than between exposure and mortality is similar to the trend reported in the HEI study (Samet et al. 2000).

Although the assessment of the relationship between PM_2.5_ and cardiovascular mortality is the main focus of this analysis, estimates of other parameters provide insights into some components of the model. For instance, the estimate of θ_0_, the baseline average ambient PM_2.5_ level over all days examined (temperature at 0°F, wind speed at 0 miles/hr), indicates that baseline ambient PM_2.5_ levels averaged approximately 9.7 μg/m^3^ over the study region from January 1999 through December 2001. The Bayesian model provides an uncertainty estimate for this parameter as well; the baseline ambient PM_2.5_ level averaged between 6.1 μg/m^3^ and 13.2 μg/m^3^ with 95% posterior probability. Some other effects to note are a positive relationship between maximum daily temperature and ambient PM_2.5_ levels (an increase of 1°F in maximum temperature is associated with an increase of 0.09 μg/m^3^ in daily average ambient PM_2.5_ level) and a negative relationship between daily average wind speed and ambient PM_2.5_ level (an increase of 1 mile/hr in average daily wind speed is associated with a decrease of 0.08 μg/m^3^ in daily average ambient PM_2.5_ level). Finally, it is of interest to examine the relationship between average ambient levels and average exposure levels in the counties of interest. The estimates of these values are presented in Table 4 along with some demographic information that was used to choose individuals for the simulator. No correlation between the demographic data and posterior mean exposure levels was observed for the seven counties in our study.

**Figure 2:**
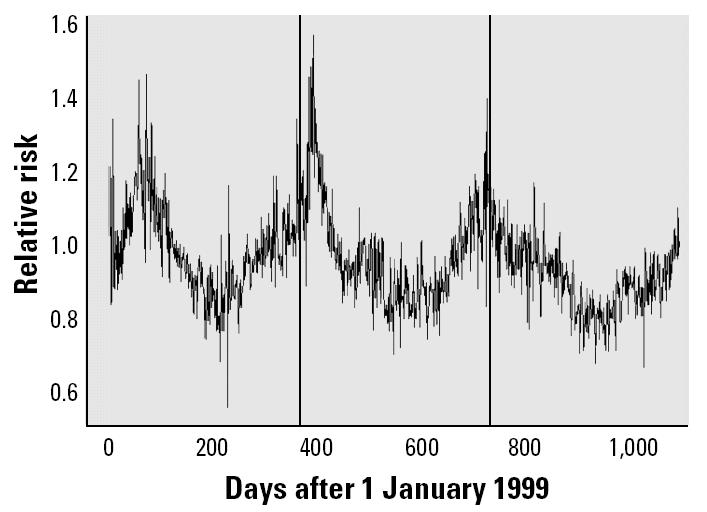
Posterior means for relative risk of mortality in Alamance County over the period studied. Vertical bars indicate 1 January for each year in the analysis.

Another interesting parameter estimated in our model is the relative risk of cardiovascular mortality in each county at each time step, λ*_ct_*. Examining the relative risk of cardiovascular mortality over the time period studied reveals some interesting patterns. All counties showed similar patterns, so we only present the results for Alamance County (Figure 2). The relative risk of cardiovascular mortality in each county follows a sinusoidal pattern that peaks when the seasonal cycle for PM_2.5_ is at its lowest point (as implied by the estimates of θ_3_ and θ_4_). The relative risk includes the influence of all of the confounding variables (maximum temperature, relative humidity, long-term cardiovascular mortality trend, and day of the week) in addition to the effect of PM_2.5_ exposure on cardiovascular mortality. Therefore, we conclude that overall cardiovascular mortality is significantly affected by numerous factors other than PM_2.5_; however, our analysis shows that PM_2.5_ exposure plays an important role in determining the relative risk of cardiovascular mortality.

### Model validation and comparison.

In order to assess whether our model gives reasonable results, we fitted different forms of the model and compared the results obtained in each case. We first considered the effect of eliminating both the spatial interpolation of ambient levels (level 1) and removing the exposure link (level 2 of our model). We call this alternate model 1. We can only fit this model in three of the seven original counties (Durham, Guilford, and Wake) because only these three counties contain at least one daily PM_2.5_ monitor. In each county, we first obtained a PM_2.5_ reading on each day by averaging the PM_2.5_ readings from all monitors that took readings on that day in the county. Prior distributions for all parameters that remain in the model (μ, β-parameters, and γ-parameters) are the same as in our full Bayesian model. We compared the results of this model with results obtained by fitting Poisson GAMs in each of the three counties individually.

The second alternate model that we fitted replaces level 2 of our Bayesian model with a simplified exposure link. Rather than including an exposure simulator, we constructed alternate model 2 by hypothesizing that exposure is equal to the ambient level plus some error [i.e., *Z_ct_* | ψ̄*_ct_*, σ*_z_*^2^ ~ *N*(ψ̄*_ct_*, σ*_z_*^2^)]. The remainder of the model is specified exactly as in our original Bayesian model. Summaries of the parameters of most interest, the β-parameters, appear in Table 5, which reports marginal posterior means and 95% credible intervals for the Bayesian models (alternate models 1 and 2) and maximum likelihood estimates with 95% confidence intervals for the classical Poisson GAMs. Note that the parameters for alternate model 2 are interpreted as the effect of a one-unit increase in PM_2.5_ exposure on the log relative risk of cardiovascular mortality, whereas the parameters in the other models relate ambient PM_2.5_ levels to the log relative risk of cardiovascular mortality.

The results from alternate model 1, the Bayesian model with no spatial interpolation or exposure link, are comparable with the results obtained by fitting the classical Poisson GAM in each of the three counties. This similarity gives evidence that the Bayesian approach produces results similar to those ordinarily obtained using the classical Poisson GAM approach. However, using a Bayesian model allows the incorporation of additional data sources and levels into the hierarchy, so the Bayesian model is more readily expanded.

As expected, the results from alternate model 2 are different from the results obtained from the classical models and alternate model 1; alternate model 2 summarizes the effect of PM_2.5_ exposure, not ambient level, on mortality. The results from alternate model 2 are more comparable with those obtained from our full Bayesian model. This similarity indicates that our model is robust to our choice of exposure simulator. However, we do not conclude that the exposure simulator is unnecessary because increased accuracy of simulated exposures will lead to more accurate estimates of the effect of exposure on mortality.

## Conclusions

By constructing a hierarchical Bayesian model that divides the process linking PM_2.5_ monitor readings and mortality into three intuitive levels, we have shown that elevated PM_2.5_ exposure is related to increased risk of cardiovascular mortality in the closely following days. We found that increases in the level of PM_2.5_ exposure are most closely related to increased relative risk of cardiovascular mortality 2 days later. In addition, we have demonstrated that the effect of increased levels of exposure on cardiovascular mortality is not equivalent to the effect of increased levels of ambient PM_2.5_ on cardiovascular mortality. Our results are similar to those reported in several studies lending additional support to our findings. In addition, we estimate that the association between ambient levels and relative risk of cardiovascular mortality on closely following days is lower than what has been previously reported in the literature.

Despite the sophistication of our model, the second level of the model leaves room for improvement. A deficiency of the second level is the absence of real exposure data. Another limitation of the second level is the simplicity of our exposure simulator; our exposure simulator ignores changes in people’s activity patterns over different days of the week and different seasons, uses fixed values to relate indoor and outdoor PM_2.5_ values, and may introduce biases in estimation by assuming that the outdoor level is the same for each individual, calculating individual exposures, and then averaging across individuals (Freedman 1999).

Future work on this type of model might focus on addressing the weaknesses in the second level of our model. For example, if real exposure data can be acquired, a data-driven version could be substituted without substantially changing the structure of the model. Similarly, a more complex exposure simulator that takes seasons and the day of the week into account could be substituted to improve the reliability of the results. Nonetheless, the results obtained by incorporating a simple exposure simulator into the model provide valuable insight into the relationship between PM_2.5_ exposure and cardiovascular mortality.

## Figures and Tables

**Table 1:** Summary of levels of hierarchical model.

Level	Data	Modeling techniques	Modeled process
1	Meteorology ambient monitor	Spatial statistical model	Spatial surface of ambient PM_2.5_ levels
2	Demographics activity patterns	Exposure simulator	Population exposure levels
3	Mortality confounders	Poisson GAM	Cardiovascular mortality

**Table 2:** Coefficients for relating ambient PM_2.5_ level to the level in indoor microenvironments.

Indoor microenvironment (*e*)	*a*_e_	*b*_e_
Residential	0.0049	0.578
Office	3.6	0.18
School	6.8	0.6
Store	9.0	0.74
Vehicle	33	0.26
Restaurant	9.8	1.0
Bar	9.8	1.0

**Table 3:** Marginal posterior summaries of several model parameters.

Parameter	Description	Mean (median)	MC error for mean	95% Credible interval
μ	Overall log RR	–0.5963 (–0.6064)	0.0651	–1.2493 to 0.07618
β_0_	Same-day mortality	0.0025 (0.0026)	0.0002	–0.0040 to 0.0092
β_1_	Lagged mortality (1)	0.0039 (0.0038)	0.0003	–0.0034 to 0.0115
β_2_	Lagged mortality (2)	0.0108 (0.0108)	0.0003	0.0028 to 0.0181
β_3_	Lagged mortality (3)	–0.0011 (–0.0010)	0.0002	–0.0078 to 0.0051
σ_z_^2^	Simulator variance	20.2853 (20.9932)	0.1489	12.3870 to 24.8422
σ_x_^2^	Monitor error	1.6495 (1.6476)	0.0009	1.5594 to 1.7457
θ_0_	Mean PM_2.5_ (μg/m^3^)	9.6856 (9.6916)	0.0275	6.1121 to 13.1849
θ_1_	Maximum temperature (°F)	0.0879 (0.0872)	0.0006	0.0224 to 0.1527
θ_2_	Wind speed (miles/hr)	–0.0799 (–0.0798)	0.0009	–0.1607 to 0.0024
θ_3_	Sine term	–0.8764 (–0.8699)	0.0061	–1.4987 to –0.2455
θ_4_	Cosine term	–1.3451 (–1.3528)	0.0091	–2.3660 to –0.3142

Abbreviations: MC, Monte Carlo; RR, relative risk.

**Table 4:** Posterior mean ambient PM_2.5_ levels and exposure levels, and demographic characteristics.

County	Ambient PM_2.5_ level (μg/m^3^)	Exposure level (μg/m^3^)	Percent male	Percent unemployed
Alamance	15.62906	13.83480	47	35
Chatham	15.64579	16.75560	48	36
Durham	15.65255	23.44071	47	34
Guilford	15.66802	28.88822	47	33
Johnston	15.61301	23.74197	48	34
Randolph	15.62650	24.23487	49	33
Wake	15.59123	12.85243	49	27

**Table 5:** Estimates of the β-parameters (credible intervals) in alternative models.

Model	β_0_	β_1_	β_2_	β_3_
Bayesian models				
Alternate model 1	–0.0025 (–0.0067 to 0.0018)	–0.0055 (–0.0106 to –0.0005)	0.0049 (–0.0001 to 0.0098)	–0.0016 (–0.0059 to 0.0025)
Alternate model 2	0.0013 (–0.0032 to 0.0057)	0.0004 (–0.0045 to 0.0054)	0.0061 (0.0013 to 0.0108)	0.0016 (–0.0028 to 0.0057)
Classical Poisson GAMs				
Durham County	–0.0036 (–0.0149 to 0.0077)	0.0024 (–0.0102 to 0.0149)	0.0124 (1.5 ×10^−6^ to 0.0248)	–0.0100 (–0.0210 to 0.0009)
Guilford County	0.0009 (–0.0084 to 0.0102)	–0.0073 (–0.0178 to 0.0033)	0.0018 (–8.5 × 10^−3^ to 0.0122)	–0.0020 (–0.0110 to 0.0069)
Wake County	–0.0032 (–0.0117 to 0.0054)	–0.0058 (–0.0152 to 0.0037)	0.0061 (–3.1 × 10^−3^ to 0.0153)	0.0050 (–0.0032 to 0.0132)
